# Beneficial Effects of Phosphite in *Arabidopsis thaliana* Mediated by Activation of ABA, SA, and JA Biosynthesis and Signaling Pathways

**DOI:** 10.3390/plants13131873

**Published:** 2024-07-06

**Authors:** Francisco Gabriel Pérez-Zavala, Jonathan Odilón Ojeda-Rivera, Luis Herrera-Estrella, Damar López-Arredondo

**Affiliations:** 1 Institute of Genomics for Crop Abiotic Stress Tolerance, Department of Plant and Soil Science, Texas Tech University, Lubbock, TX 79409, USA; francisco.perez@ttu.edu (F.G.P.-Z.); joo29@cornell.edu (J.O.O.-R.); luis.herrera-estrella@ttu.edu (L.H.-E.); 2 Unidad de Genómica Avanzada/Langebio, Centro de Investigación y de Estudios Avanzados del IPN, Irapuato 36821, Mexico

**Keywords:** *Arabidopsis thaliana*, phosphite, abscisic acid, jasmonic acid, salicylic acid, phosphorus sensing, defense responses, stress conditions

## Abstract

Phosphite (Phi) has gained attention in agriculture due to its biostimulant effect on crops. This molecule has been found to benefit plant performance by providing protection against pathogens, improving yield and fruit quality as well as nutrient and water use efficiency. It is still unclear how Phi enhances plant growth and protects against multiple stresses. It has been hypothesized that Phi acts by directly affecting the pathogens and interacting with the plant cellular components and molecular machinery to elicit defense responses. This study elucidates the mechanisms underlying Phi’s beneficial effects on plants, revealing their complex interplay with fundamental signaling pathways. An RNA-seq study of *Arabidopsis* seedlings under optimal and limiting phosphate conditions helped us unveil Phi’s role in promoting plant growth by activating the expression of the genes involved in the biosynthesis and signaling pathways associated with abscisic acid (ABA), salicylic acid (SA), and jasmonic acid (JA). The expression of ABA-related genes, known for their involvement in stress response and development regulation, is triggered by Phi treatment, contributing to enhanced resilience and growth. Simultaneously, the activation of the SA pathway, associated with defense responses, suggests Phi’s potential in bolstering plant immunity. Moreover, Phi influences JA biosynthesis and signaling, which are crucial for defense against herbivores and pathogens, thereby strengthening plants’ defenses. Our findings reveal a multifaceted mechanism through which Phi benefits *Arabidopsis* development. Understanding its intricate interplay with key signaling pathways opens avenues for leveraging Phi as a strategic tool to enhance plant resilience, immunity, and growth in agricultural and ecological contexts.

## 1. Introduction

Fertilizers are applied to supplement the essential nutrients required for optimal crop growth and productivity. Early farmers empirically recognized the value of fertilization by applying livestock manure to improve crop yields in the Neolithic land around 8000 years ago [1]. Then, superphosphate made of bones and phosphate rock was crucial in sustaining crop productivity during the 19th century [2]. The next significant advancement in crop fertilization came when the Haber–Bosch process was developed, which allowed the massive and economic use of atmospheric nitrogen for fertilization. Then, multiple Nitrogen–Phosphorus–Potassium (NPK) fertilizer formulations and improved crop seeds allowed for extensive and intensive cultivation with superior yields.

More recently, fertilization, combined with beneficial elements and biostimulant molecules, has arisen as a tool for sustainable agriculture with the potential to counter the effects of climate change and new emergent pathogens while improving yields by up to 33.6% [3]. Phosphite (Phi) is a biostimulant with proven beneficial effects in many plant species (see [4] for a review). Phi is a reduced chemical form of P that shares structural similarities with orthophosphate (Pi), the primary P source for most plants [5]. Although most plants cannot metabolize it [6,7], the foliar application of Phi has been shown to protect plants from pathogens, including multiple species of the genus *Phytophthora*, as well as protozoa, oomycetes, fungi, bacteria, and nematodes [8]. The pathogen protective effect of Phi has been demonstrated in over 20 crops, including avocado and citrus trees [9,10,11,12,13,14,15]. This has led to the broad commercialization of Phi, mainly as a fungicide, thus opening a global market of about $107 million in 2023 only for potassium phosphite [6,14,16]. Multiple reports on other economically relevant crops, such as wheat, banana, tomato, and pepper, have claimed the beneficial effects of Phi application on yield, dry mass, chlorophyll, amino acid and protein content, and more recently, on nutrient use efficiency and tolerance to water deficit [17,18,19,20,21].

The mechanism by which Phi elicits such beneficial effects on plants is still unclear and has been proposed to occur through two separate mechanisms of action. The first mechanism involves the direct impact of Phi on the pathogen, as it can allosterically inhibit enzymes that use Pi as a substrate, affecting the synthesis of compounds indispensable to the cell structure and physiology [15]. The second mechanism is related to the potential of Phi to activate the expression of defense response genes that enhance plant tolerance to biotic and abiotic stresses. It has been proposed that Phi stimulates systemic acquired resistance (SAR)- and abscisic acid (ABA)-related pathways [22,23]. While studies showing the beneficial effects of Phi are relatively abundant, the exact mechanism of action of Phi and how it activates these defense response pathways have yet to be elucidated.

Because of its structural similarity with Pi, Phi can interact with the same cellular components and molecular machinery as Pi. Both Phi and Pi ions are absorbed by plants using the same protein transporters, leading to competition when both ions are present in the soil solution [7,24]. Consequently, in agricultural settings, maintaining the proper P levels in plants is essential when using Phi to prevent negative impacts on plant growth and yield [25]. Researchers have leveraged Phi’s structural similarity to Pi to study how plants respond to Pi scarcity and to better understand Phosphate Starvation Responses (PSRs). Although Phi is structurally and behaviorally similar to Pi in terms of absorption and transport, plants cannot use Phi as a P source, so Phi is not a metabolic analog of Pi. Nonetheless, plants perceive Phi as Pi through what is likely the Pi receptor in plants. This perception disrupts the activation of PSRs in Pi-deprived plants, such as root hair elongation, lateral root development, anthocyanin accumulation, and the activation of many Pi-starvation-responsive genes, including Pi transporters [24,26,27,28].

The impact of Phi depends on the plant’s phosphorus status; it is beneficial when plants grow with sufficient Pi levels but detrimental when Pi is scarce. To understand the molecular mechanisms underlying the effects of Phi on plants, this study examines the phenotype and transcriptional landscape of *Arabidopsis thaliana* seedlings in response to Phi treatment under optimal and limiting Pi conditions. Our findings indicate that Phi primes plants to respond to multiple biotic and abiotic stresses mediated by abscisic acid (ABA), salicylic acid (SA), and jasmonic acid (JA) pathways.

## 2. Results

### 2.1. Phi Suppresses Pi Starvation Responses and Positively Affects Arabidopsis Growth under Optimal Pi Conditions

**Figure 1:**
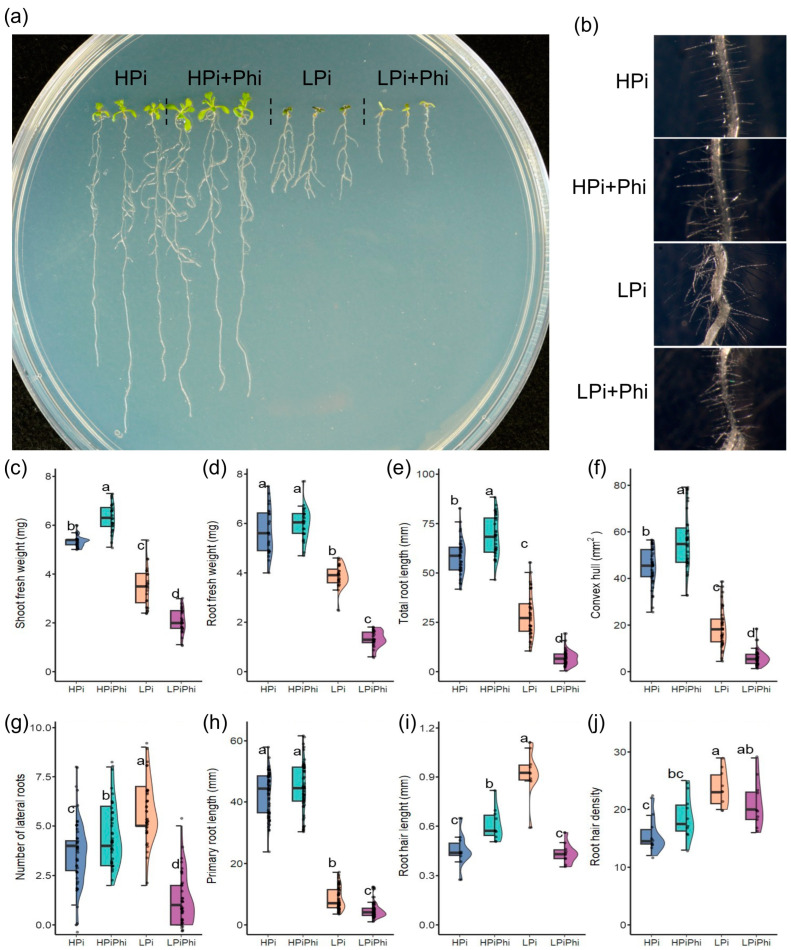
Effects of phosphite (Phi) on *Arabidopsis thaliana* on different levels of Phosphate (Pi). (**a**) Representative photographs of 10 dag *Arabidopsis* plants under different phosphite and phosphate treatments. (**b**) Representative photographs of 10 dag *Arabidopsis* hair roots under different phosphite and phosphate treatments, (**c**) Shoot fresh weight (*n* = 24, each data point is the average of ten plants), (**d**) Root fresh weight (*n* = 24, each data point is the average of ten plants) (**e**) Total root length (*n* = 40), (**f**) Convex hull area (*n* = 40), (**g**) Lateral root count (*n* = 40), (**h**) Principal root length (*n* = 60), (**i**) Hair root length (*n* = 10, each data point is the average of 10 roots hairs, each data point was taken from a different plant), (**j**) Root hair density (*n* = 10). HSD tests were performed, and different letters mean significant differences. Panels (**c**–**h**): half of the data points were taken randomly from data from two different and independent experiments.

Based on the biostimulant effects of Phi on the growth and development of over 20 crops, we evaluated the effect of Phi on *Arabidopsis* seedlings at the phenotypic and molecular levels. *Arabidopsis* seedlings were grown under optimal (HPi) and low phosphate (LPi) conditions with or without Phi and evaluated 10 days after germination (dag) (Figure 1a). These experiments used two Phi concentrations as follows: 50 μM (LPi + Phi) to treat Pi-starved seedlings and 250 μM (HPi + Phi) to treat plants growing under optimal Pi levels. These Phi concentrations were chosen based on previous reports and our experience, which suggested that higher Phi concentrations might cause phytotoxic effects, which would confound the analysis of the transcriptional responses. A series of parameters, including shoot and root fresh weight, primary root length, and lateral root number, among others (see Materials and Methods), were determined to investigate the effect of Phi treatment.

We found that under LPi conditions, the treatment with 50 mM Phi severely reduced shoot and root growth (Figure 1). In Pi-deprived seedlings, 50 mM Phi also attenuated the formation of lateral roots and the accumulation of anthocyanins, which are characteristic components of the PSR in *Arabidopsis* [29,30]. Phi negatively impacted the metrics associated with the root architecture, such as lateral root number, primary root length, root weight, total root length, and convex hull (Figure 1e–j). Under LPi conditions, Phi inhibited root hair elongation by 52.7% and reduced hair root density by 59.7%. These results show that Phi directly attenuates the PSR in *Arabidopsis* at the phenotypic level. Our results align with those previously reported [28]. By contrast, treatment with 250 mM Phi of seedlings grown under Pi sufficiency enhanced the plant’s fresh weight by 17.9% for shoots and 5.46% for roots compared to the untreated control (Figure 1c,d). Phi treatment in HPi conditions enhanced elongation and the density of root hairs by 20.6% and 61.1%, respectively (Figure 1b,i,j). These results align with previous reports on the effect of Phi on stimulating root growth under optimal fertilization conditions [18].

### 2.2. Phi induces Transcriptional Changes in Arabidopsis under Both Low and Optimal Pi Levels

To investigate the molecular mechanisms underlying the effects of Phi on *Arabidopsis* seedlings under LPi and HPi conditions, we performed RNA-seq analysis to evaluate differences in overall mRNA abundance between treatments. We produced the following three biological replicates per the treatments established above: HPi, LPi, HPi + Phi, and LPi + Phi, with a total of 12 RNA-seq libraries (see Materials and Methods). Quality and pseudo-alignment statistics of the different RNA-seq libraries are presented in Tables S1 and S2. The multidimensional scaling (MDS) exploratory analysis showed little dispersion among the libraries, and they clustered by treatment as expected (Figure S1). HPi and HPi + Phi libraries were much closer to each other than LPi to LPi + Phi. This suggests that the Phi treatment has a bigger impact on the transcriptional landscape of Pi-deprived plants (LPi) than those growing under P sufficiency (HPi).

**Figure 2:**
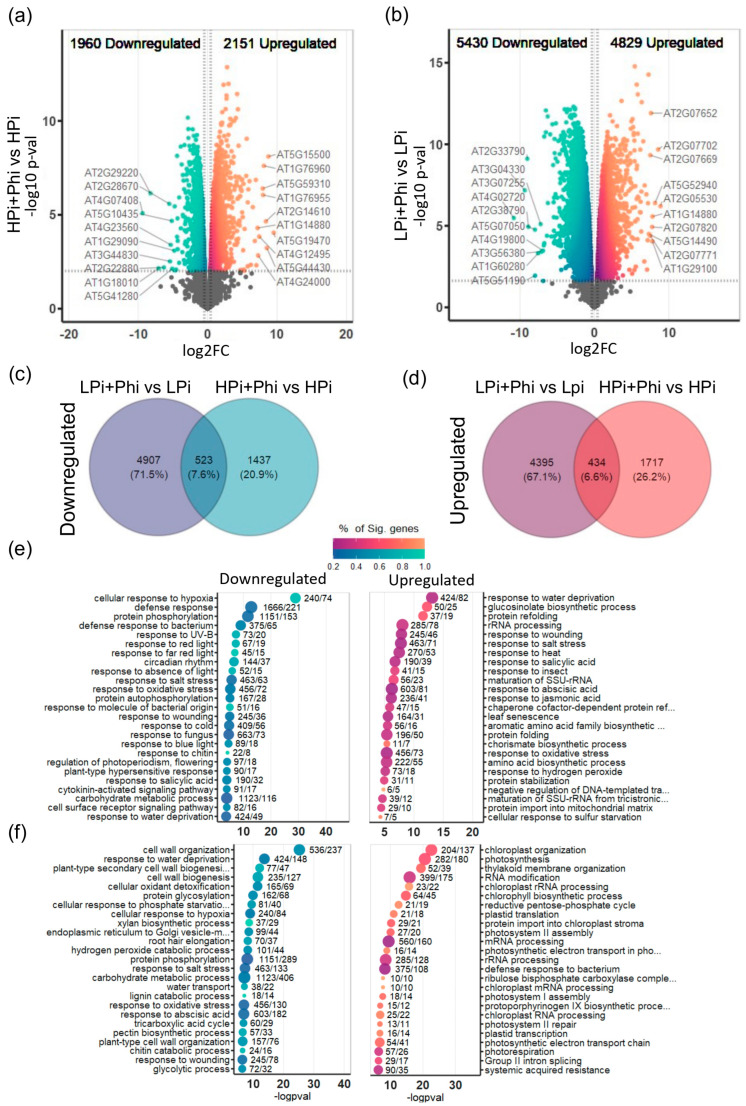
Transcriptional landscape modification by phosphite treatments under different Phosphate (Pi) levels (**a**) Volcano plot of all the differentially expressed genes (DEGs) in High Phosphate and phosphite combined treatment (+Pi + Phi) relative to High Phosphate treatment (+Pi). Upregulated DEGs are presented in orange, in blue are the downregulated DEGs, and the top 10 genes ranked according to fold-change are highlighted. (**b**) Same as (**a**) but for the treatment LPi + Phi relative to LPi. (**c**) Shared and unique downregulated DEGs in both contrasts. (**d**) Shared and unique upregulated DEGs in both contrasts. (**e**) Enriched Terms of Gene Ontology of the Up- and Down-regulated genes of (**a**). Enriched Terms in the Upregulated DEGs are presented in orange, in blue are the enriched terms of the downregulated DEGs. (**f**) Is the same as (**e**) but of (**b**) DEGs.

We then performed quasi-likelihood F-tests to determine the differentially expressed genes (DEGs) between treatments and *t*-tests relative to a threshold (±20% expression) to control the false discovery rate (FDR) [31]; benchmarks of this method suggest that it improves on controlling FDR more than previous methods like just setting an FDR cutoff. Comparison of the transcriptomes of LPi and the HPi plants without Phi treatment resulted in 4715 upregulated genes and 4028 downregulated genes in response to Pi starvation (Table S3). Gene Ontology (GO) analysis of DEGs revealed enriched GO terms in the upregulated DEGs related to PSR, such as “cellular response to phosphate starvation”, “response to abscisic acid”, “root hair elongation”, “response to oxidative stress”, and “phenylpropanoid biosynthetic process” (Figure S2; Table S4). Regarding downregulated DEGs, GO terms enriched were mostly related to photosynthesis (Figure S2; Table S4). This proves that the Pi treatments functioned as expected. To determine the effects of Phi treatment on global transcript accumulation, the treatment conditions HPi + Phi and LPi + Phi were compared against their respective controls, HPi and LPi. Relative to HPi conditions, we found 2151 upregulated and 1960 downregulated DEGs under the HPi + Phi treatment (Figure 2a; Table S5).

Relative to the LPi conditions, we found 4829 upregulated and 5430 downregulated DEGs in the LPi + Phi treatment (Figure 2b; Table S6). This corroborates that Phi treatment has a more drastic effect on Pi-deprived seedlings than those growing under P sufficiency. A Venn diagram helped us to determine that a common set of 523 DEGs are downregulated and 434 DEGs upregulated by Phi in both treatments (Figure 2c,d; Table S7). These results provide evidence that a common set of responses is activated by Phi, independent of the P status of the plant.

### 2.3. Phi Activates Plant Defense Responses under Pi Sufficiency

To determine the biological processes that underlie *Arabidopsis* responses to Phi, we performed GO enrichment analysis of the upregulated and downregulated DEGs for each treatment. Relative to the HPi conditions, GO analysis of upregulated genes in the HPi + Phi condition showed that the top 25 significant enriched GO categories are related to abiotic and biotic stresses. For instance, we found “response to water deprivation”, “glucosinolate biosynthetic process”, “response to salt stress”, “response to heat”, “response to salicylic acid”, “response to wounding”, “response to abscisic acid”, “response to oxidative stress”, “response to jasmonic acid”, and “response to insect” (Figure 2e). Other enriched GO categories for the upregulated DEGs in the HPi + Phi treatment were also related to stresses (“defense response to virus” and “response to nematode”), nutrient utilization (“response to iron ion starvation”, “sulfate assimilation”, and “cellular response to phosphate starvation”), and C metabolism (“response to glucose” and “starch catabolic process”) (Table S8). The top 25 significantly enriched GO terms in the downregulated genes of the HPi + Phi treatment relative to the HPi condition were related to plant response to light and circadian rhythm (“response to red light”, “regulation of photoperiodism flowering”, “circadian rhythm”, and “response to far-red light”), and diverse stresses (“cellular response to hypoxia”, “defense response to bacterium”, “defense response”, “plant-type hypersensitive response”, “response to UV-B”, “response to molecule of bacterial origin”, “response to salt stress”, and “response to salicylic acid”) (Figure 2e). More GO categories enriched and statistically significant in the downregulated genes of the HPi +Phi treatment was also related to biotic and abiotic stresses, as presented in Table S8.

Interestingly, several GO terms were commonly enriched in both the upregulated and downregulated terms of the HPi + Phi conditions, including the “response to abscisic acid”, “response to salt stress”, “response to oxidative stress”, “response to salicylic acid”, “response to water deprivation”, “response to wounding”, “carbohydrate transmembrane transport”, “response to cold”, “response to light stimulus”, “detoxification”, and “response to hydrogen peroxide” (Table S8). Moreover, we found that some GO-enriched categories in up and downregulated DEGs have related terms. For example, “cellular response to cold” and “response to cold” are enriched in down and upregulated DEGs. Also, “xenobiotic detoxification” and “detoxification” are enriched in down and upregulated DEGs, respectively, as well as “response to salicylic acid” and “systemic acquired resistance”, enriched in the upregulated and downregulated DEGs. System-acquired resistance is known to be activated by SA. These data suggest that under Pi sufficiency and Phi treatment, different components of the plant defense responses are up or downregulated, likely to prevent premature activation of the defense mechanisms and avoid growth penalties. Also, these results suggest a possible priming effect of Phi on the plant defense responses.

Phi suppressed the phenotypic changes commonly elicited in response to Pi starvation in our study (Figure 1). Therefore, we investigated how Phi interferes at the molecular level with those responses. We determined the enriched GO categories of upregulated and downregulated DEGs in LPi + Phi treatment relative to the LPi condition. Among the top 25 enriched upregulated GO terms, we found 15 terms related to chloroplast and photosynthesis, including “chloroplast organization”, “photosynthesis”, “thylakoid membrane organization”, “chloroplast rRNA processing”, “chlorophyll biosynthetic process”, “plastid translation”, “protein import into chloroplast stroma”, “chloroplast mRNA processing”, “photosystem I assembly”, “chloroplast RNA processing”, “protoporphyrinogen IX biosynthetic process”, “plastid transcription”, “photosystem II repair”, “photosynthetic electron transport chain”, and “photorespiration” (Figure 2f). As shown in Table S9, other statistically significant GO terms enriched in this analysis were also related to photosynthesis. This suggests that Phi prevents photosynthesis from shutting down in response to Pi starvation. Among the most significatively enriched GO categories of upregulated DEGs in the LPi treatment relative to HPi, 56 out of 100, were found as downregulated in the LPi + Phi vs. LPi comparison, suggesting that Phi downregulates the expression of a large subset of low-Pi-responsive genes. Among the GO terms that Phi downregulates in Pi-deprived seedlings, we found typical categories activated in the PSR like “response to oxidative stress”, “cell wall organization”, “cellular response to phosphate starvation”, “root hair elongation”, “phenylpropanoid biosynthetic process”, and “phosphate ion transport” (Figure 2f; Table S9).

Because the Phi treatment in optimal Pi conditions enhances shoot growth, we further explored the possible mechanisms that will help explain this effect. We searched for GO categories representing biological processes that may enhance nutrient uptake and remobilization of internal and external resources for plant growth. We found the enrichment of several GO terms related to mineral nutrition and C metabolism, e.g., “cellular response to phosphate starvation”, “cellular response to sulfur starvation”, “response to iron ion starvation”, “sulfate assimilation”, “starch catabolic process”, “response to glucose”, “starch catabolic process”, “deoxyribonucleotide catabolic process” “tyrosine catabolic process”, “deoxyribose phosphate catabolic process”, and “L-serine catabolic process” (Figure 2e). These enriched GO terms suggest that multiple nutrient transporters and nutrient recycling genes are upregulated in response to Phi. We identified 46 nutrient transporter-encoding genes upregulated in HPi plants treated with Phi (Figure S3). These genes included *NITRATE TRANSPORTER 1* (AT1G12110), *NITRATE TRANSPORTER 2* (AT1G08090), *DEGRADATION OF UREA 3* (AT5G45380), *XYLULOSE 5-PHOSPHATE/PHOSPHATE TRANSLOCATOR* (AT5G17630), *PHOSPHATE TRANSPORTER 1;4* (AT2G38940), *PHOSPHATE TRANSPORTER 4;2* (AT2G38060), *PHOSPHATE TRANSPORTER 3;1* (AT5G14040), *PHOSPHOENOLPYRUVATE/PHOSPHATE TRANSLOCATOR* (AT5G33320), along with 37 more related to Fe, S, B, Mg, Na, and Zn transport (Figure S3a; Table S10). We also found upregulated genes related to organic acid secretion in HPi plants treated with Phi (Figure S3b; Table S10), suggesting that Phi treatment may enhance shoot biomass accumulation by boosting nutrient transporters and secretion of organic acids that might help the plant scavenge resources from the media or remodel the interaction with the soil microbiome.

### 2.4. Phi Enhances the Biosynthesis of ABA, JA, and SA, and Shows a Priming Effect on the Associated Signaling Pathways

**Figure 3:**
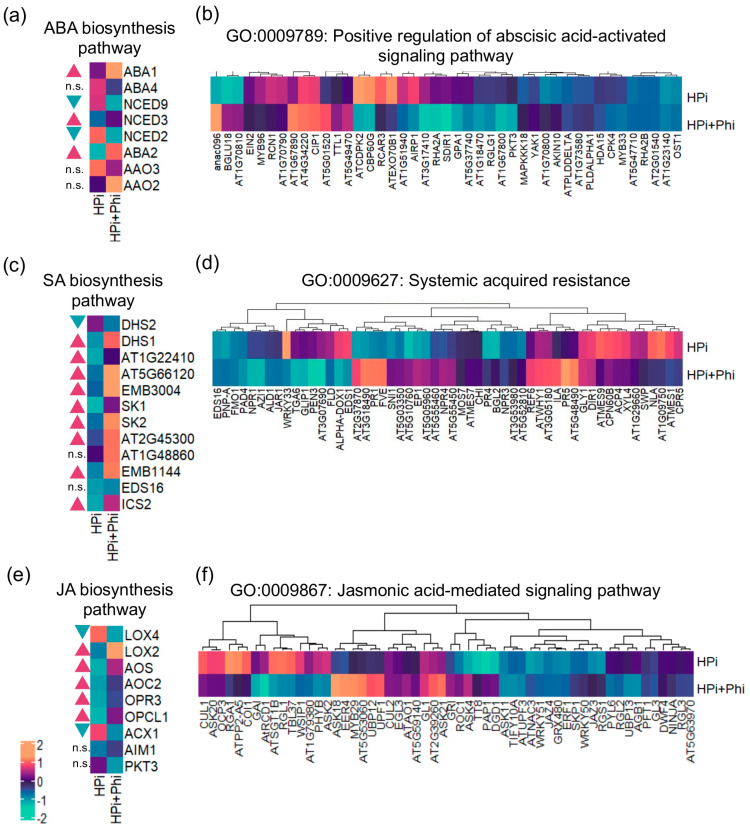
Effect of phosphite treatments on the transcription of different hormones. (**a**) Heatmap of genes of the ABA biosynthesis pathway. (**b**) Heatmap of the GO term “positive regulation of abscisic acid-activated signaling pathway”. (**c**) Heatmap of DEGS related to salicylic acid biosynthesis. (**d**) Heatmap of the GO term “systemic acquired resistance”. (**e**) Heatmap of DEGs related to jasmonic acid biosynthesis. (**f**) Heatmap of the GO Term “Jasmonic acid-mediated signaling pathway”. Genes of the biosynthetic pathways were taken from the Plant Metabolic Network database. For all plots: Blue triangles represent downregulated genes, red triangles represent upregulated genes, and n.s. stands for non-significant. Likelihood ratio tests according to the edgeR pipeline were performed on each pair of genes to determine if there are significant differences between treatments.

ABA, JA, and SA are key in regulating plant responses to biotic and abiotic stresses and diverse developmental processes. To better understand the effect of Phi on genes belonging to GO categories related to these plant hormones (Figure 2e; Table S8), we further investigated the expression patterns of genes involved in their biosynthesis and associated signaling pathways. The ABA biosynthetic pathway requires four enzymatic steps catalyzed by six enzymes, neoxanthin synthase (ABA4), 9-cis-epoxycarotenoid dioxygenase (NCED9, NCED3, NCED6, and NCED2), xanthoxin dehydrogenase (ABA2), and abscisic aldehyde oxidase (AAO3) [32,33]. In addition, an antheraxanthin epoxidase/zeaxanthin epoxidase (ABA1) synthesizes violaxanthin, the ABA precursor [34]. We found that the expression of three genes involved in the key enzymatic steps of ABA biosynthesis is significantly upregulated in HPi + Phi conditions (Figure 3a). However, some DEGs in the GO category “positive regulation of abscisic acid-activated signaling pathway” are downregulated in response to the same treatment (Figure 3b). This gene set includes 43 genes, from which we can highlight the *SUCROSE NON-FERMENTING-1-RELATED PROTEIN KINASE 2–6* (*SNRK2–6*/AT4G33950), *REGULATORY COMPONENTS OF ABA RECEPTOR 3* (*RCAR3*/AT5G53160), *MITOGEN-ACTIVATED PROTEIN KINASE KINASE KINASE 18* (*MAPKKK18*/At1g05100), and *MYB DOMAIN PROTEIN 96* (*MYB96*/AT5G62470), which are well-known to participate in the ABA signaling processes.

SA biosynthesis involves six enzymatic reactions carried out by 3-deoxy-7-phosphoheptulonate synthase (DHS1/AT4G39980, DHS2/AT4G33510, and AT1G22410), 3-dehydroquinate synthase (AT5G66120), 3-dehydroquinate dehydratase/shikimate 5-dehydrogenase (MEE32/AT3G06350), shikimate kinase (SK1/AT2G21940 and SK2/AT4G39540), and 3-phosphoshikimate 1-carboxyvinyltransferase (EPSP/AT2G45300 and AT1G48860), to synthesize the SA precursor, chorismate, and isochorismate synthase (ICS1/AT1G74710, ICS2/AT1G8870) that produces isochorismate, which is the last enzymatic step required for SA synthesis. Up to 9 of the 11 genes encoding these enzymes are significantly upregulated under the HPi + Phi treatment (Figure 3c). SA is known to activate systemic acquired resistance in plants. We found that the transcripts of several genes that serve as molecular markers of disease in plants and SA-induced responses, such as *PATHOGENESIS-RELATED 1* (*PR1*/AT2G14610), *PATHOGENESIS-RELATED 2* (*PR2*/AT3G57260), *PATHOGENESIS-RELATED 4* (*PR4*/AT3G04720), and *PATHOGENESIS-RELATED 5* (*PR5*/AT1G75040), are also elevated in the Phi-treated plants (Figure 3d).

In the case of JA-biosynthesis genes, we found that four genes that encode for enzymes catalyzing crucial steps for the JA build-up, *LIPOXYGENASE 3* (*LOX3*/AT1G17420), *LIPOXYGENASE 2* (*LOX2*/AT3G45140), *ALLENE OXIDE SYNTHASE* (*AOS1*/AT5G42650), and *OPC-8:0 COA LIGASE 1* (*OPCL1*/AT1G20510), were significantly upregulated under HPi + Phi (Figure 3e). On the other hand, *MYC2* (*MYC2*/AT1G32640), a core component of the JA signal transduction, was downregulated (Table S5). A more detailed analysis of the expression pattern of JA signaling pathway genes revealed that some are downregulated and that the upregulated genes like *ASK18* and *MYB29* are negative regulators of this signaling pathway (Figure 3f).

Altogether, our data show that the Phi treatment in Pi-sufficient plants enhances the biosynthesis of these three plant hormones. However, the associated signaling mechanisms are tightly regulated and are only partially activated or not activated at all, likely to avoid having severe effects on plant growth. This may result in quicker plant response once the stress is perceived while avoiding growth penalties in the absence of stress.

### 2.5. Phi Suppresses Local and Systemic Responses to Pi Starvation

**Figure 4:**
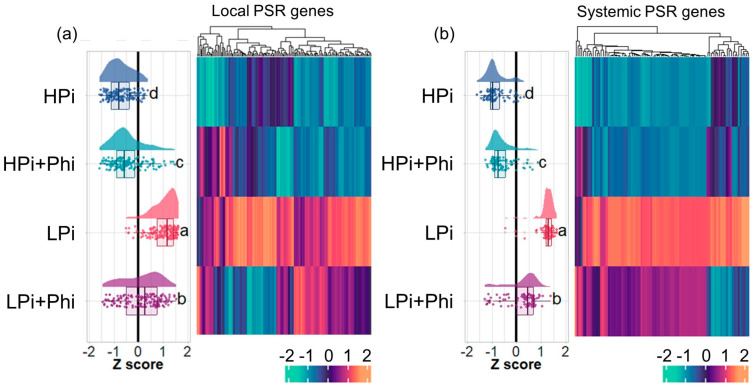
Effect of phosphite (Phi) on local and systemic PSRs. (**a**) Heatmap of the local PSRs genes. (**b**) Heatmap of the systemic PSRs. Tukey’s multiple comparisons of means test were performed between treatments; different letters mean significant differences between treatments.

In plants, the PSRs operate mainly through two well-defined regulatory systems, namely the systemic response, which tightly regulates sets of genes in response to internal Pi levels, and the local response, which controls genes in response to external Pi levels and regulates root development and architecture independently of the internal Pi content [35]. We investigated whether Phi treatment affects the transcript accumulation of gene sets involved in the *Arabidopsis* local and systemic responses to Pi starvation [35]. We found that of 301 genes of the local PSR systems, 153 were significantly upregulated by LPi relative to HPi, of which 89 (58.1%) were significantly downregulated by Phi in Pi-deprived seedlings (Figure 4a; Table S11). Of the 110 systemic PSR genes, 90 had higher transcript levels in our LPi vs. HPi analysis, of which 82 (91.1%) were significantly downregulated by Phi in Pi-deprived seedlings (Figure 4b; Table S12). These results correlate with the enrichment of GO categories “cellular response to phosphate starvation” and “phosphate ion transport”, which were enriched as downregulated in LPi + Phi condition (Figure 2f). In these GO categories, we found genes typically activated in response to Pi starvation, e.g., many high-affinity phosphate transporters and *PHOSPHATE TRANSPORTER TRAFFIC FACILITATOR 1* (*PHF1*/AT3G52190), *SPX DOMAIN GENE 1* (*SPX1*/AT5G20150), *SPX DOMAIN GENE 2* (*SPX2*/AT2G26660), *SPX DOMAIN GENE 3* (*SPX3*/AT2G45130), and *PHOSPHATE DEFICIENCY RESPONSE 2* (*PDR2*/AT5G23630) (Figure 2f; Table S6). Interestingly, for the same set of 153 local PSR genes, we found that Phi treatment upregulates 29 genes (18.9%) and downregulates 12 (7.84%) in HPi conditions. In the case of the 90 systemic PSR genes, Phi treatment upregulates 23 genes (25.5%) and downregulates only 4 (4.4%). This suggests that Phi activates some PSRs even in optimal Pi levels and downregulates them at low Pi levels, adding evidence of the duality of Phi effects depending on Pi levels (Tables S11 and S12).

### 2.6. Phi-Dependent Shutdown of STOP1 and PHR1 Signaling Pathways

**Figure 5:**
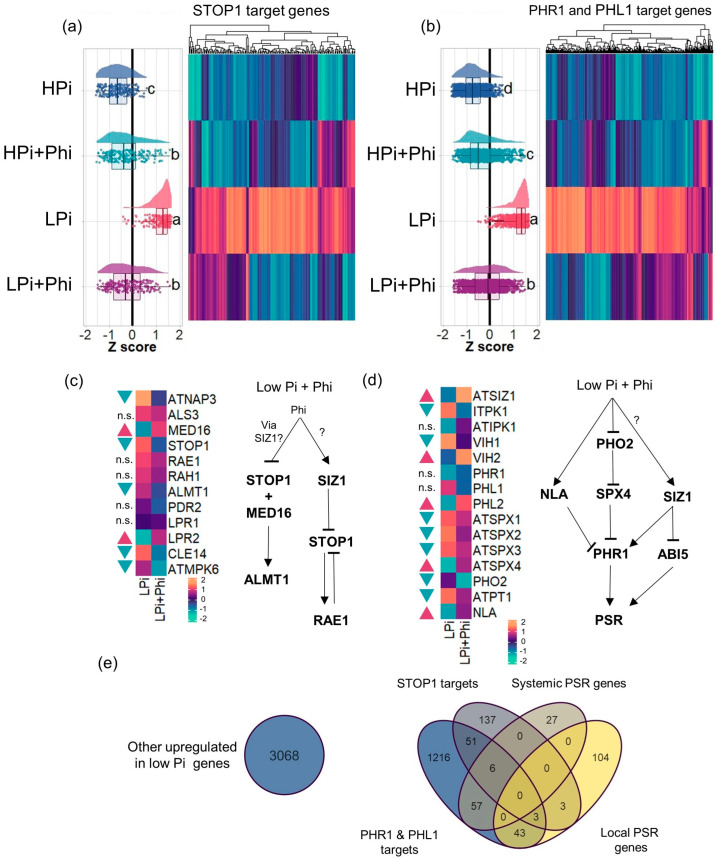
Effect of phosphite (Phi) on STOP1 and PHR1/PHL1 targets. (**a**) Raincloud plot and heatmap of the STOP1 targets. (**b**) Raincloud plot and heatmap of the PHR1/PHL1 targets. (**c**) Changes in gene expression of STOP1 signaling pathway related genes. (**d**) Changes in gene expression of PHR1 signaling pathway related genes. Question marks in (**c**,**d**) represent putative or still unknown regulatory components of the pathway. (**e**) Venn diagram of the genes related to PHR1/PHL1, STOP1, Systemic PSR, and local PSRs, including genes that are not part of these sets. For (**a**,**b**): Tukey´s multiple comparisons of means test were performed between treatments; different letters mean significant differences between treatments. In (**c**,**d**): blue triangles represent downregulated genes and red triangles represent upregulated genes, n.s. stands for non-significant. Likelihood ratio tests according to the edgeR pipeline were performed on each pair of genes to determine if there are significant differences between treatments.

PHR1, with its homologs PHL1 and STOP1, are the main transcription factors (TFs) that orchestrate *Arabidopsis’s* systemic and local PSRs. Target genes of PHR1, PHL1, and STOP1 have been previously identified in *Arabidopsis* [36,37]. We investigated whether Phi attenuates the expression of PHR1, PHL1, and STOP1 targets in LPi conditions [36,37]. We found that Phi has a generalized attenuation effect on the expression of PHR1, PHL1, and STOP1 target genes and does not have a specific effect over any gene set specifically under the control of these TFs. In the case of STOP1, from its predicted 1279 targets, we found that 200 were significantly upregulated in the LPi treatment. Of these 200 low-Pi-responsive genes, the expression of 143 (71.5%) was significantly reduced by Phi under LPi conditions (Figure 5a; Table S13). From the 5306 predicted targets of PHR1 and PHL1, the transcript level of 1376 was significantly higher in Pi-deprived seedlings compared to the controls. Treatment of Pi-deprived seedlings with Phi reduced the transcript level of 1027 genes (74.6%) of these low-Pi-responsive genes. (Figure 5b; Table S14). Because in the case of local and systemic responses, Phi treatment upregulated some PSR genes in plants grown under sufficient Pi conditions, we decided to test if this was also the case for STOP1, PHR1, and PHL1 targets. Phi treatment upregulated 46 STOP1 target genes (23%) and 187 PHR1 target genes (13.6%) under HPi conditions (Table S14).

Because Phi represses transcript accumulation of STOP1 and PHR1–PHL1 target genes under LPi, we analyzed the expression pattern of STOP1, PHR1, and the associated signaling pathways in detail to determine at which level Phi intervenes and blocks them. In the case of the STOP1-related pathways, four important components have been identified, which are STOP1-MED16 (regulated by RAE1 and RAH1), PDR2, ALS3-STAR1, and MPK6 [29]. We found that Phi downregulates the expression of *STOP1*, *MPK6*, *STAR1,* and other components that act downstream in the local Pi response, including *CLE14* and *ALMT1* (Figure 5c), whereas *PDR2* and *RAE1* remain unchanged. It has been reported that ALMT1-mediated malate exudation results in a Fe redistribution that activates a redox cycle that leads to meristem exhaustion in Pi-deprived seedlings. This redox cycle, in turn, activates the expression of *CLE14*, which is directly involved in meristem exhaustion.

To test if Phi treatment influences meristem exhaustion, we examined the meristem of Pi-deprived seedlings in the presence and absence of Phi using the cell cycle and quiescent center (QC) marker lines, CycB1,1::*uidA* and QC46::*uidA*, respectively. We found a marked increase in the activity of the reporter gene of CycB1,1::*uidA* plants starting 7 dag in plants grown under HPi with and without Phi treatment. In contrast, CycB1;1::*uidA* activity drastically diminished 11 dag in plants grown under LPi, but maintained in Pi-deprived plants treated with Phi (Figure S4). In the case of the QC46::*uidA* reporter line grown under Pi-starvation (LPi), GUS expression was completely lost 9 dag (Figure S5). This correlated with dramatic changes in the meristematic region, which was highly vacuolated, and the QC and initial cells were not clearly defined. When Pi-deprived plants were grown in the presence of Phi, the expression of QC46::*uidA* was still observed 11 dag, suggesting the QC cells kept their identity and activity, similarly to when the plants were grown under Pi sufficiency (Figure S5). These data suggest that Phi delays meristem exhaustion under LPi conditions, possibly by targeting molecular components of the local Pi-sensing system that activate this process.

In the case of the PHR1-related signaling pathway, we found that the negative post-transcriptional regulators of PHR1, including *NLA* and *SPX4*, are upregulated in response to Phi under LPi (Figure 5d). Interestingly, we discovered that *SIZ1* is upregulated under LPi seedlings treated with Phi. *SIZ1* encodes a SUMO E3 ligase that has been reported to have crucial regulatory roles in the PSR in plants. SIZ1 is a PHR1 activator that, in turn, deactivates STOP1 and negatively regulates the TF abscisic acid sensitive 5 (ABI5). ABI5 is a central regulator of the ABA signaling pathway controlling multiple physiologic processes, which has recently been linked to the activation of the PSR [38]. Altogether, these data suggest that Phi interferes with the signaling pathways controlling the PSR at multiple levels. It opens the possibility that Phi targets perhaps an upstream genetic component that commonly regulates local and systemic signaling transduction pathways, such as the Pi sensor system(s), or a closely related component.

**Figure 6:**
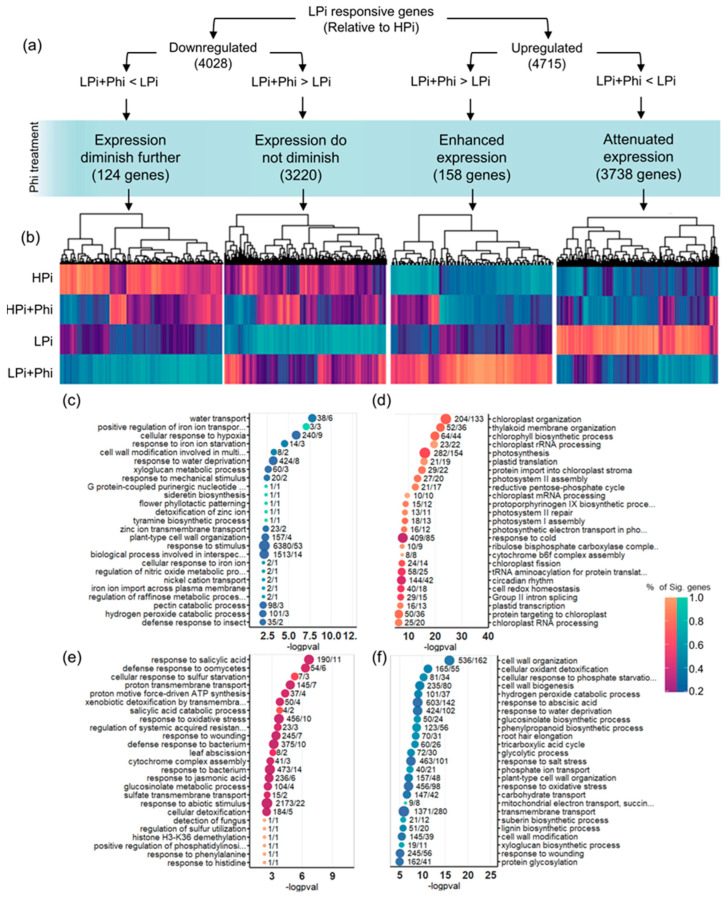
Effect of phosphite (Phi) on the DEGs of the LPi treatment relative to the HPi. (**a**) Flow diagram describing how the gene filtering was performed. Expression levels of all genes that changed expression in the LPi + Phi treatment in reference to the LPi treatment relative to HPi were taken. Quasi likelihood F test was performed to determine if the change was significant. (**b**) Heatmap of the genes filtered in (**a**). (**c**) Enriched GO terms of the DEGs that are downregulated by LPi relative to HPi genes that have further diminished expression in the LPi + Phi treatment. (**d**) Enriched GO terms of the DEGs that are downregulated and activated by LPi relative to HPi genes did not diminish their expression in the LPi + Phi treatment. (**e**) Enriched GO terms of the DEGs that are activated by LPi relative to HPi genes that have enhanced expression in the LPi + Phi treatment. (**f**) Enriched GO terms of the DEGs that are activated by LPi relative to HPi that have attenuated expression in the LPi + Phi treatment.

A Venn analysis was used to determine further the effect of Phi on the *Arabidopsis* transcriptional responses to Pi (Figure 5e; Tables S11–S14). We found that the four data RNA-seq sets related to the low Pi response (local PSR, systemic PSR, PHR1 + PHL1 targets, and STOP1 targets) comprise 1647 (34.9%) of the 4715 upregulated DEGs in response to Pi-deprivation. The remaining 3068 (65.1%) upregulated DEGs contained enriched GO categories related to response to oxidative stress, cell wall modifications, and osmotic stress (Table S15; Figure S6). Other significantly enriched in the top 100 GO terms were “response to abscisic acid”, “response to 1-aminocyclopropane-1-carboxylic acid” (the ethylene precursor), and “response to jasmonic acid”, which are hormones that have been previously found to be also crucial for the PSRs [38,39,40]. This new gene set of low-Pi-responsive genes is also affected by the Phi treatments. From 3068 genes activated during LPi, 2211 (72.1%) were downregulated in the Phi treatments (Figure S6). In total, from the 4714 upregulated genes in the LPi treatment relative to HPi, the Phi treatment reduced the transcript level of 3738 genes (79.3%) and enhanced the expression of 158 genes (3.4%) (Figure 6). In the case of 4028 downregulated genes in Pi-deprived plants, the transcript level of only 124 (3.1%) was further decreased by Phi treatment. These numbers show that Phi significantly impacts the accumulation of transcript of most upregulated PSR genes, but not downregulated, and appears to affect all signaling pathways (Figure 6).

### 2.7. Phi-Dependent Shutdown of STOP1 and PHR1 Signaling Pathways

A Venn analysis of the DEGs sets of LPi vs. HPi, LPi + Phi vs. HPi, and HPi + Phi vs. HPi contrasts helped us further dissect the responses of *Arabidopsis* to Phi. We found a core of 421 genes is common between all these gene sets (Figure S7; Table S16), which, according to the enriched GO terms, are related to JA, ABA, and SA. We also found that “glucosinolate biosynthesis process” is upregulated in the three treatments. The GO term “cellular response to phosphate starvation” is part of this common backbone even when Phi treatment is applied to seedlings grown under Pi sufficiency (Figure S7; Table S17). The enriched terms unique to LPi vs. HPi are GO terms related to the common PSR responses, downregulated under Phi treatment. The DEGs unique to LPi + Phi vs. HPi are more related to biotic defense responses comprising systemic acquired resistance, innate immune responses, and defense responses to bacteria and fungi (Figure S7; Table S17). This indicates that Phi enhances plant defense responses activated in LPi and activates additional ones. The unique GO categories in the HPi + Phi relative to HPi are related to heat, salt, water, light stresses, and nematode response (Figure S7; Table S17).

## 3. Discussion

### 3.1. Phi Has a Positive Effect on Plant Growth

Phi significantly enhanced *Arabidopsis* shoot fresh weight by 11.4% in HPi conditions (Figure 1c). In line with previous reports that demonstrate that Phi significantly improves crop root growth [18,19,41], we observed that the Phi treatment under HPi conditions significantly enhances the number of lateral roots (31.4%), root total length (20.1%), the convex hull of the root (26.1%), and the length of hair roots (33.3%). Phi increased root biomass and induced changes in root architecture that can impact nutrient uptake. Likewise, other mechanisms, such as the activation of nutrient transporters, also lead to directly enhanced nutrient uptake, thus working to stimulate plant growth. Our results of RNA-seq analysis revealed that when growing under Pi sufficiency, Phi treatment increases the accumulation of transcripts of genes involved in the uptake, recycling, and solubilization of several nutrients. Phi treatment increased transcript levels of 46 nutrient transporters, including those involved with major essential nutrients (N, P, K) and other nutrients such as Fe, S, B, Mg, Na, and Zn (Figure S3), which along with the enhanced root traits could further boost plant nutrition. Although we did not measure nutrient content in our experiments, these data correlate with the reported enhanced nutrient content in several crops upon Phi treatment [42,43,44,45,46].

Apart from the upregulation of nutrient transporters, other GO categories related to catabolism, such as “starch catabolic process”, “response to glucose”, “deoxyribonucleotide catabolic process”, “L-serine catabolic process”, and “deoxyribose phosphate catabolic process”, were enriched in HPi + Phi treatment relative to HPi (Table S5). Phi appears to enhance CO_2_ assimilation by activating genes related to starch degradation. Mutations on starch catabolic genes have a negative impact on yield [47], suggesting that Phi enhances starch degradation to enhance plant growth. Starch degradation has also been demonstrated to have a positive impact on plants under abiotic stresses, which could help maintain plant growth under drought or high salinity [48] and protect them from the effects of osmotic stress [49].

To determine whether Phi alters gene expression independently of the Pi status in the plant, we assessed whether there is a common set of responses between Phi in different Pi conditions. While Phi diminishes the expression of most PSR genes, it activates the expression of some genes independently of the Pi status of the plant. We discovered that Phi increases the transcript level of a common set of genes in LPi + Phi and HPi + Phi conditions. This set of genes showed enriched GO terms for “response to jasmonic acid”, “response to abscisic acid”, and “response to salicylic acid” (Figure S5 and Table S18). These findings support the observation that Phi enhances plant tolerance to pathogen infection and abiotic stresses such as drought and high salinity.

Phi might benefit plant performance and nutrient use efficiency, ultimately having the potential of positively impacting yield. Phi may act as a protective shield and help enhance plant growth and resilience to multiple stress conditions. The improved growth may be an effect of sugar and starch degradation, as shown in Table S5, that may have the pleiotropic effect of enhancing drought responses, as these molecules act as osmolytes to protect plants [48,50]. These results align with the Phi effects reported in wheat, soybean, and eucalyptus, in which Phi enhanced drought tolerance [18,21,51], probably by activating ABA responses (Figure 2 and Figure S7, and Table S5).

### 3.2. Phi Activates Defense Responses against Multiple Biotic and Abiotic Stresses Using ABA, SA, and JA Signaling Pathways

We found that Phi treatment significantly influences several gene ontology (GO) terms, such as “response to water deprivation”, “glucosinolates biosynthetic process”, “response to salt stress”, “response to heat”, “response to salicylic acid”, “response to insects”, “response to abscisic acid”, and “response to jasmonic acid” (Figure 2e). These are among the top 25 significant responses triggered by Phi. Additionally, in the top 100 enriched GO terms, we identified “response to osmotic stress”, “response to nematodes”, and “response to viruses” (Table S8). These terms cover a variety of stresses that plants may encounter.

Previous research has shown that Phi activates genes associated with plant hormones such as ABA, ethylene (ET), SA, and JA. These hormones play a key role in enhancing plant tolerance to multiple stresses. To better understand how Phi influences plant defense responses, we tracked the biosynthesis and signaling pathways of ABA, SA, and JA (Figure 2e; Table S8). We discovered that ABA, SA, and JA biosynthetic genes were significantly upregulated in plants treated with Phi (Figure 3a,c,e), indicating an increase in the associated signaling pathways. However, contrary to expectations, we did not observe significant upregulation of genes involved in “positive regulation of abscisic acid-activated signaling pathway”, “systemic acquired resistance”, and “jasmonic acid signaling pathway” (Figure 4b,d,f). This suggests that Phi activates only certain genes related to stress responses but not the entire signaling pathways. The precise mechanism that allows plants to avoid activating these signaling pathways without stress detection remains unknown, though epigenetic modifications may play a role. It has been reported that Phi downregulates miRNAs that control genes related to ABA, JA, ET, SA, and auxin (AUX) post-transcriptionally [52]. Our findings suggest these pathways are tightly regulated to prevent unnecessary energy expenditure and growth penalties, which would affect plant fitness. Chemical priming with Phi could enable plant defense mechanisms to be activated more rapidly and effectively to stress conditions, enhancing their tolerance.

Our study revealed the significant upregulation of *ABI1* and *ABI2*, which are known to inhibit SnRK2 kinases [53,54], which play a crucial role in ABA signal transduction pathways, and their absence can disrupt the signaling circuit [55]. To counteract the impact of ABI1 and ABI2 on SnRK2 kinases, the ABA perception system suppresses these inhibitors but only in the presence of ABA. We also observed the upregulation of *PYL3*, a component of the ABA sensor, which suggests the activation of the ABA signaling pathway. However, we found no significant increase in ABA pathway activity (Figure 3a). Furthermore, our data showed the upregulation of *APF* (AT1G69260), a negative regulator of ABI5. This implies that although the perception of ABA is present, the final trigger of the ABA response, ABI5, is inactivated due to APF action. Regarding the JA signaling pathway, while some elements were upregulated in the HPi + Phi treatment, MYC2, a critical component of the JA signaling pathway, was downregulated (Table S5). This suggests that the JA pathway is not fully activated and remains on standby until a pathogen challenges the plant. This tight regulation of ABA and JA likely helps prevent negative impacts on plant growth and development, as both hormones have been shown to inhibit plant growth when present at high levels [56,57]. In contrast, our findings indicate that the SA biosynthetic pathway and signaling pathways are active, with upregulated DEGs and marker genes for SA and pathogens such as *PR1*, *PR2*, and *PR5* (Table S5). This suggests that the plant is partially or fully utilizing the SA pathway, unlike the ABA and JA pathways that are only primed. Prior studies on SA treatments in plants suggest a positive effect of SA even in non-stress conditions [58,59,60]. This may explain the enhanced defense and growth observed in plants treated with SA. Additionally, SA is a negative regulator of ABA and JA responses, indicating that plants could use SA to regulate other hormone responses.

Phi is already commercially used to combat fungal diseases in many plants. In tomato, potato, spinach, soybean, grapevine, and blue lupin, Phi application activated genes related to SA responses or SA biosynthesis [61,62,63,64,65,66,67,68,69]. In some of these cases, Phi also activated responses related to JA, ET, and brassinosteroids. The Phi-activation defense mechanisms seem similar in all plants and related to SA-activated responses and biosynthesis.

### 3.3. Phi Effects on Low Pi Conditions Are Mainly Caused by Suppressing the Activation of the PSRs

In the case of LPi conditions, we found that Phi has a negative impact on shoot and root growth (Figure 1). This effect has been previously reported [25,43,44,70]. The negative effect of Phi on the growth of Pi-starved plants is mainly caused by its capacity to suppress the PSR, which reduces Pi uptake and prevents the activation of the pathways that mediate P recycling. Our results demonstrate that Phi affects 79.3% of low-Pi-responsive genes, including genes related to the GO terms “cellular response to phosphate starvation” and “phosphate ion transport.” The GO term “cellular response to phosphate starvation” is enriched in downregulated DEGs, supporting the hypothesis that the negative impact on plant growth in LPi conditions is due to the effect of Phi preventing the activation of PSRs.

Low Pi availability triggers the development of more lateral roots and increases the density and length of root hairs. However, the application of Phi inhibits these processes (Figure 1), affecting root weight, total root length, lateral root number, and the convex hull. These changes negatively affect nutrient uptake. Our transcriptomic analysis supports these findings. The LPi + Phi treatment, compared to LPi alone, showed enrichment in downregulated genes associated with root growth and modifications. These include GO terms such as “cell wall organization”, “plant-type secondary cell wall biogenesis”, “pectin biosynthetic process”, “cell wall biogenesis”, “root hair elongation”, and “lignin catabolic process” (Figure 2f; Table S9). These results suggest that Phi inhibits the cell wall remodeling process that is activated in Pi-deprived seedlings.

Interestingly, it is likely that Phi treatment further blocks the low Pi signaling pathways. Phi modulates the local Pi signaling pathway by increasing the expression of *SIZ1*, a SUMO E3 ligase, that targets STOP1, preventing its coupling with MED16 [71,72], a STOP1 co-activator [73]. In line with these results, genetic components of this signaling pathway, like *CLE14*, *ALMT1*, and *MPK6* [29], are also downregulated by Phi. Therefore, at the biological level, the treatment with Phi of a plant growing under LPi, will prevent the activation of phenotypic and molecular mechanisms that help the plant thrive under such conditions. Thus, an adequate internal P status is crucial to promote plant growth and fitness by applying Phi.

The idea that Phi, a molecule that attenuates the PSRs in multiple plants [26,61,74], also activates some PSRs seems counterintuitive. However, aside from our results, this effect has also been reported for alfalfa [75], suggesting that this may be the pathway that Phi affects, leading to the activation of SA, JA, ABA, and other plant hormone pathways. Being plant hormones the orchestrators, this Phi-mediated mechanism may be similar in all plants (Figure 6 and Figure S7).

### 3.4. Phi Modulates the Local and Systemic Responses to Low Pi

We evaluated the effect of Phi on the expression of previously reported gene sets involved in local and systemic PSRs [35]. We found that in LPi conditions, applying Phi reduced the expression of both systemic and local PSR genes. Specifically, 61.4% of systemic PSR genes and 91.1% of local PSR genes showed decreased expression levels (Figure 4). This correlates well with the observation that Phi diminishes the expression of genes targeted by STOP1 and PHR1 (Figure 5). These results strongly suggest that Phi targets the receptors activating local and systemic responses upstream of PHR1 and STOP1. Since the systemic receptor is responsive to the internal status of Pi and the local sensor is responsive to the external level of Pi present in the soil solution, our data suggest that it binds to an intracellular sensor(s) that regulates systemic Pi responses and to another sensor(s) likely located in the cell membrane that perceives the external concentration of Pi. In the biological context, these findings reveal the nature of the P-sensing system in plants. Further research by taking advantage of the non-metabolizable nature of Phi might help us gain more details on the putative local and systemic sensors.

### 3.5. Practical Implications under Field Conditions

In an agricultural context, the potential benefits of Phi application represent an important advantage for cultivating crops. By positively impacting root growth, nutrient use, carbon metabolism, and stress responses, Phi-activated benefits might expand to make more rational use of resources, positively impacting yield and benefiting farmers and the environment. The fact that Phi activates a common set of genes related to JA, ABA, and SA regardless of the plant P status opens new opportunities to design more effective practices for soils with low P availability. Importantly, the levels of Pi and Phi in field conditions need to be optimized based on the crop of interest, agricultural practices, soil physicochemical properties, and environmental conditions. Engineered plants able to metabolize Phi as the only P source have been developed and shown to allow more environmentally friendly agricultural schemes by improving P use and outcompeting grass and broadleaved weeds [76,77]. Integrating Phi into current agricultural schemes or combined with engineered plants might allow for the optimization of fertilizer and pesticide use globally.

Although this study was conducted with *Arabidopsis,* a model plant, under controlled conditions, it provides a valid framework to assess Phi’s effects on economically important crops, considering the findings reported here. Importantly, multiple studies have reported the beneficial effects of Phi in diverse plant species, which suggest that Phi-associated effects and responses are generalized to plants. Nevertheless, based on the crop of interest, many factors should be considered in those studies, e.g., the levels of optimum and deficient Pi, maximum levels of Phi application to prevent toxicity, and optimal levels of Phi to maximize benefits to the crop. The implications of our findings go beyond just clarifying how Phi acts inside the plant; they open avenues for leveraging Phi as a strategic tool to enhance plant resilience, immunity, and growth in agricultural and ecological contexts.

## 4. Conclusions

Our experiments revealed that Phi treatment benefits *Arabidopsis* growth by promoting shoot and root development under high Pi (HPi) conditions. The positive impact of Phi on plant growth can be largely attributed to its influence on root architecture, increased expression of genes encoding nutrient transporters, and activation of defense response genes. These modifications lead to more efficient nutrient uptake and greater resilience to environmental stresses. Interestingly, many genes that benefit plant growth under Phi treatment are also activated by Pi starvation, suggesting that synthetic biology strategies could potentially be employed to activate the signaling pathways to enhance both defense responses and Pi uptake. By leveraging these pathways, it may be possible to improve plant health and productivity in different growing conditions. Furthermore, our results indicate that Phi targets both systemic and local Pi systems, providing an opportunity to use this Pi analog as a tool to identify mutants affected by Pi sensing. This approach could lead to a deeper understanding of Pi sensing and signaling mechanisms, potentially informing future agricultural practices and contributing to crop improvement efforts.

## 5. Materials and Methods

### 5.1. Biological Material and Plant Growth

*A. thaliana* ecotype Col-0 (CS70000) and the transgenic lines QC46::*uidA* [78] and CycB1;1::*uidA* [79] were used for this study. *Arabidopsis* seeds were surface-sterilized by soaking them in 100% ethanol for seven minutes, then in a commercial bleach solution (sodium hypochlorite 7.5%) diluted up to 0.2× (sodium hypochlorite 1.5% final concentration) for seven minutes; the seeds were rinsed three times with sterile distilled water and then kept in water in the dark at 4 °C for two days for stratification. *Arabidopsis* seeds were sown onto 1% agar 0.1× Murashige and Skoog (MS) medium plates. The agar was washed three times with Milli-Q water to remove potential soluble P traces and other soluble compounds that may be naturally present. We used monopotassium phosphate (KH_2_PO_4,_ Pi) (Sigma Aldrich, St. Louis, MO, USA, CAS. No. 7778-77-0, 99%) or monopotassium phosphite (KH_2_PO_3,_ Phi) (Wanjie International Company Ltd., Hangzhou, China, CAS. No. 13977-65-6, 98%) as required. Pi was used for propagation purposes. Seedlings were grown in a Percival chamber at 22 °C and a 16 h light/8 h dark photoperiod with a PAR intensity of ≈135 µmol·m^−2^·s^−1^.

### 5.2. Evaluation of Phi Effect on Arabidopsis Shoot and Root Growth

To study the effect of Phi on *Arabidopsis* growth, two treatment conditions were established, 50 µM Phi added to the low P condition determined by the lack of Pi in the media (LPi, 0 Pi) and 250 µM Phi added to the high P condition containing 1 mM Pi (HPi). Both LPi and HPi were set up as controls. Phi was added directly to the MS medium, and the pH was adjusted to 5.7. The plants were photographed 10 days after germination (dag), and principal root length, total root length (including secondary and tertiary roots), lateral root count, convex hull, and other parameters were evaluated using the software Root Nav v1.8.1.0 [80]. *Arabidopsis* shoots and roots were separated, and their fresh weight was recorded as a pool of 10 individuals to weigh the little mass of *Arabidopsis* seedlings. Therefore, each data point is presented as an average of 10 seedlings.

The Phi concentrations used in this work, 50 µM and 250 µM Phi combined with 0 Pi and 1 mM Pi, respectively, were selected based on previous reports and our experiments. In the case of the LPi + Phi (0 Pi and 50 µM Phi) treatment, a higher Phi concentration might cause severe phytotoxic effects because no Pi is present in the media. This effect would be more severe due to the washed agar we utilized, from which Pi traces and other minerals were removed. This toxicity would confound the analysis of the Phi-specific transcriptional responses. Therefore, a low Phi dose, enough to observe the attenuation of the Pi-starvation response at the phenotype and molecular levels in *Arabidopsis* was selected for this treatment. In the case of the HPi + Phi (1 mM Pi and 250 µM Phi) treatment, a higher Phi concentration would strongly compete with Pi, as it has been suggested that due to their structural similarities, both are taken up via the same protein transporters. Therefore, to avoid such competition of Phi with Pi, which might lead to negative effects on the plant, 250 µM Phi was selected to study the beneficial effects of Phi supplementation under high Pi levels.

### 5.3. Experiments for RNA-seq Studies

Under the established treatments, 200 seedlings were harvested per condition at 10 dag. Three biological replicates per treatment were generated and named as follows: HPi for optimal Pi, -Pi for 0 Pi, HPi + Phi for optimal Pi treated with 250 µM Phi, and LPi + Phi for 0Pi treated with 50 µM of Phi. The 200 seedlings per treatment were collected and pooled to make a biological replicate, then flash-frozen in liquid nitrogen and homogenized to isolate total RNA using TRIzol (Invitrogen, Carlsbad, CA, USA). mRNA-seq libraries were generated using the TruSeq Illumina protocol for the pool of 200 seedlings from each of the different treatments. Libraries were sequenced using a NovaSeq 6000 platform with paired-end 150 bp reads at Novogene Corporation Inc. (Sacramento, CA, USA).

### 5.4. Data Checks and RNA-seq Data Analysis

Quality of sequencing reads was performed using TrimGalore v0.6.6 [81], which is a wrapper of FastQC v0.11.9 [82], and cutaddapt v3.5 [83] to remove adapters and low-quality reads. The read abundance was computed using the pseudo-alignment program kallisto v0.46.2 [84]. The official release of the *A. thaliana* genome annotation, Araport11 (https://www.arabidopsis.org/ (accessed on 11 September 2023)) [85], was used to build the index file for pseudoalignment. To integrate the counts from kallisto into downstream analysis, the R package tximport v1.20.0 [86] was used. Differential expression analysis was performed with R using the edgeR package v3.34.0 [87,88]. The pipeline was the same as reported by [89] but using the quasi-likelihood F-tests [90,91] instead of the chi-squared approximation to the likelihood ratio statistic, resulting in more conservative and rigorous type I error rate control. After the quasi-likelihood F-tests to determine differentially expressed genes (DEGs) between treatments, we used *t*-tests relative to a threshold to control false discovery rate (FDR) [31]; benchmarks of this method suggest that it improves on controlling FDR more than previous methods like just setting an FDR cutoff. The threshold for selecting differentially expressed genes with this method was a log fold change of 0.263, an increase or decrease in expression of 20%. The code developed for this study can be found at https://github.com/Fperezzavala/Phosphite-RNAseq_arabidopsis (accessed on 20 May 2024). The common Biological Coefficient of Variation (BCV) was 0.1235 for our data.

Gene Ontology (GO) [92] enrichment analysis was carried out using the R package topGO v2.44.0, using the “elim” algorithm [93]. Relations between sets of DEGs were analyzed using the R package dplyr v1.0.7 [94] and visualized with the R packages ggvenn v0.1.9 [95] and ggplot2 v3.3.5 [96].

### 5.5. Histochemical Analysis

GUS activity was determined using the complete *Arabidopsis* seedlings incubated with GUS reaction buffer (5-bromo-4-chloro-3-indolyl glucuronide) at 37 °C overnight. The X-Gluc solution consisted of 0.5 mg/mL 5-bromo-4-chloro-3-indolyl glucuronide in a solution containing NaHPO_4_ 100 mM (pH 7.0), β-mercaptoethanol 10 mM, EDTA 10 mM, n-lauryl-sarcosine 0.1%, Triton X-100 0.1%, and potassium ferry- and ferrocyanide 5 mM, to catalyze oxidation reactions [97]. Subsequently, the plantlets were fixed and cleared using the method described by [98]. At least 15–20 plantlets were analyzed per condition and genotype, and representative seedlings were imaged under Nomarski optics (LEICA DMR) to identify the expression pattern of the *uidA* reporter gene in the meristematic region of the primary root.

## Data Availability

All raw data generated in this work are deposited at the National Center for Biotechnology Information (NCBI) BioProject ID PRJNA1073557. Targets of evaluated TF used in this work are available in [35,36,37]. All the biosynthetic pathways were taken from the Plant Metabolic network, available at: https://www.plantcyc.org/ (accessed on 7 February 2024) [99]. The raw data are available at https://www.ncbi.nlm.nih.gov/bioproject/PRJNA1073557 (accessed on 5 February 2024)

## Supplementary Materials

The following supporting information can be downloaded at: https://www.mdpi.com/article/10.3390/plants13131873/s1, Figure S1. Multidimensional scaling (MDS) plot constructed on normalized counts of the RNA-seq libraries. Figure S2. Transcriptional landscape of *Arabidopsis* seedlings under phosphate starvation (LPi), and Pi starvation treated with phosphite (LPi + Phi). Figure S3. Effect of phosphite (Phi) on the expression of nutrient acquisition-related genes. Figure S4. Effect of phosphite (Phi) on mitotic activity of the meristematic region of *Arabidopsis* CycB1;1::*uidA* seedlings. Figure S5. Effect of phosphite (Phi) on the activity of quiescent center (QC) of *Arabidopsis* QC46::*uidA* seedlings. Figure S6. Effect of phosphite (Phi) on the expression pattern of non-STOP1/PHR1 target genes or genes previously described as part of the local or systemic PSRs that are upregulated in low phosphate (LPi) treatments. Figure S7. Effect of phosphite (Phi) on the transcriptional profile of *Arabidopsis* under the different treatments relative to the control with high phosphorus level (HPi). Table S1. Sequencing run information. Table S2. Kallisto run information. Table S3. Differentially expressed genes in LPi treatment relative to HPi. Table S4. Enriched Gene Ontology (GO) terms in the up and downregulated differentially expressed genes (DEGs) in LPi treatment relative to HPi. Table S5. Differentially expressed genes in HPi + Phi treatment relative to HPi. Table S6. Differentially expressed genes in LPi + Phi treatment relative to LPi. Table S7. Shared genes between HPi + Phi and LPi + Phi up and downregulated genes. Table S8. Enriched GO terms in the up and downregulated differentially expressed genes in HPi + Phi treatment relative to HPi. Table S9. Enriched GO terms in the up and downregulated differentially expressed genes in LPi + Phi treatment relative to LPi. Table S10. Genes with increased expression in HPi + Phi relative to HPi. Table S11. Effect of phosphite on local phosphate starvation response (PSR) genes. Table S12. Effect of phosphite on systemic upregulated phosphate starvation response (PSR) genes. Table S13. Effect of phosphite on STOP1 targets genes. Table S14. Effect of phosphite on PHR1/PHL1 targets genes. Table S15. Effect of phosphite on phosphate starvation response (PSR) genes found in this study that are not part of STOP1/PHR1/PHL1 or local and systemic responses. Table S16. Dissection of the intersection of genes upregulated in LPi, LPi + Phi, and HPi + Phi. Table S17. Enriched GO terms of the intersection of genes upregulated in LPi, LPi + Phi, and HPi + Phi.
