# Supplementary material for: Beneficial Effects of Phosphite in Arabidopsis thaliana Mediated by Activation of ABA, SA, and JA Biosynthesis and Signaling Pathways
Source: Plants (Basel). 2024 Jul 6;13(13):1873. doi: 10.3390/plants13131873 (PMC11244317; doi:10.3390/plants13131873)
Supplement: Supplementary file 1 [file plants-13-01873-s001.zip › plants-3056516-supplementary.pdf]

Supporting Information

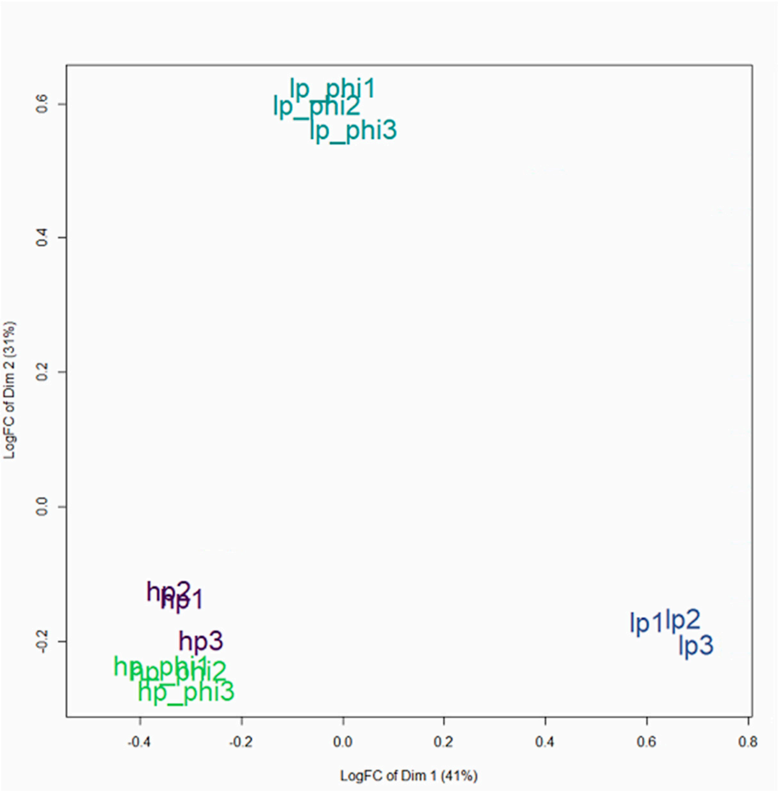

Figure S1. Multidimensional scaling (MDS) plot constructed on normalized counts of the RNA-seq libraries. Labels represent the three replicates (1,2, 3) of the different treatments: high phosphate (hp, purple), high phosphate + phosphite (hp_phi, green), low phosphate (lp, blue), low phosphate+ phosphite (lp_phi, cyan).

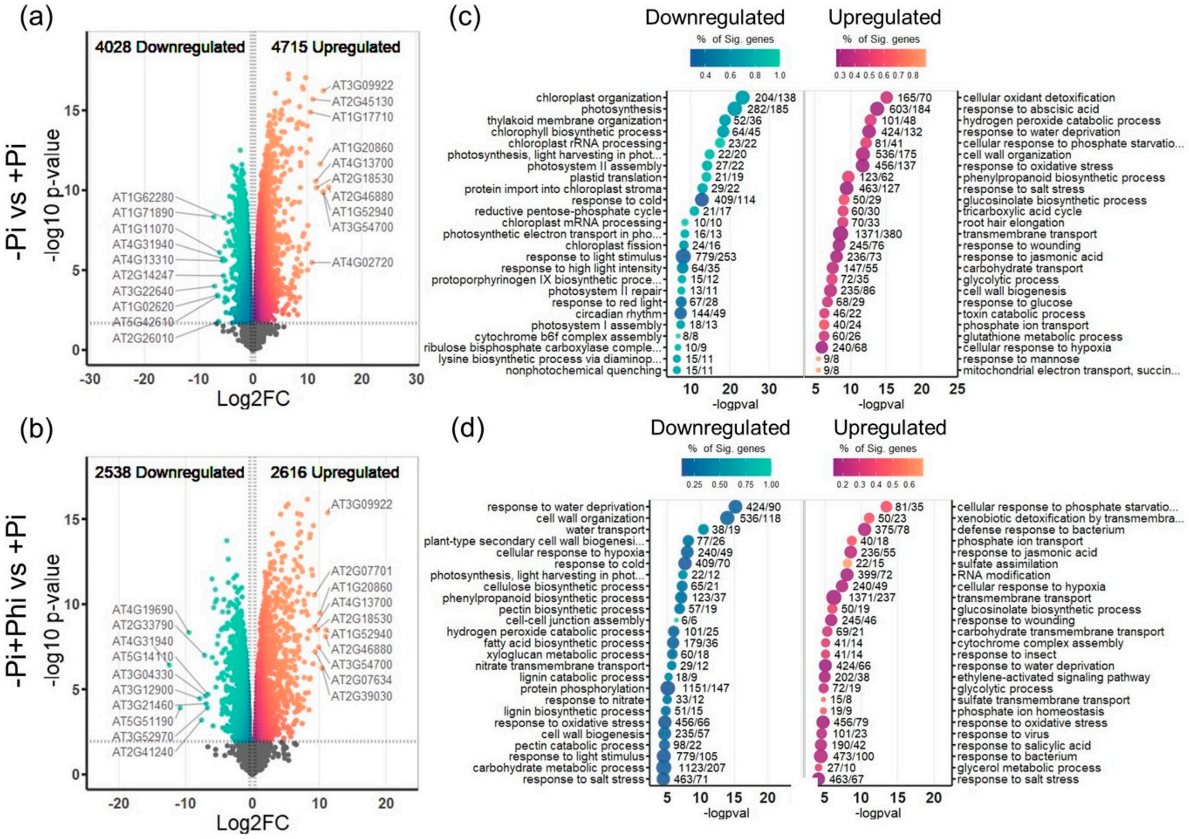

Figure S2. Transcriptional landscape of *Arabidopsis* seedlings under phosphate starvation (LPi), and Pi starvation treated with phosphite (LPi+Phi). Volcano plot of all the differentially expressed genes (DEGs) in (a) LPi and (b) LPi+Phi relative to high Pi control (HPi). Gene Ontology (GO) enriched terms of DEGs in (c) a and (d) b. The top 10 genes ranked according to fold-change are highlighted in a and b.

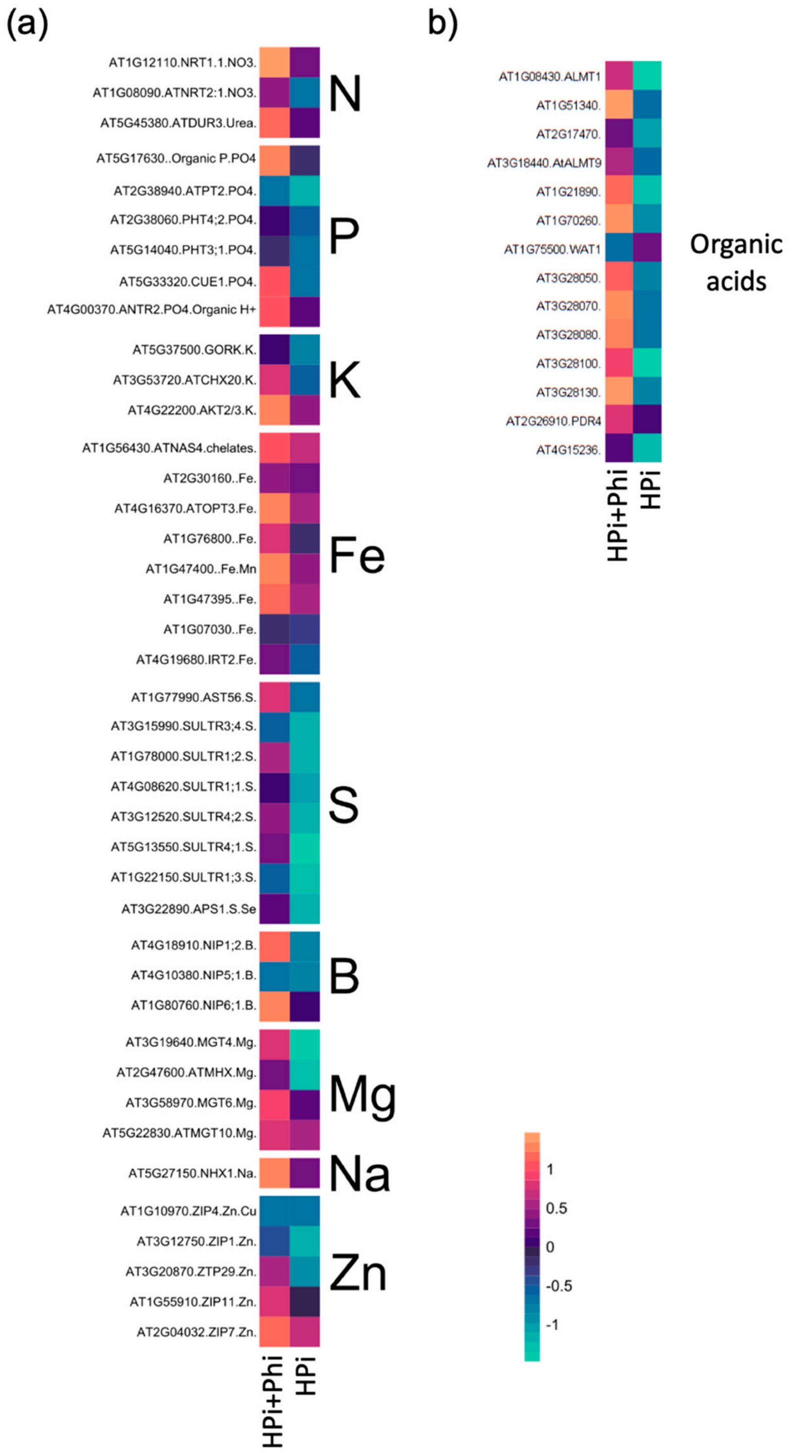

Figure S3. Effect of phosphite (Phi) on the expression of nutrient acquisition-related genes. heatmap showing the expression level (z-score) of genes encoding transporters of (a) macro and microelements, and (b) transporters of organic acids. All heatmaps share the same color scale.

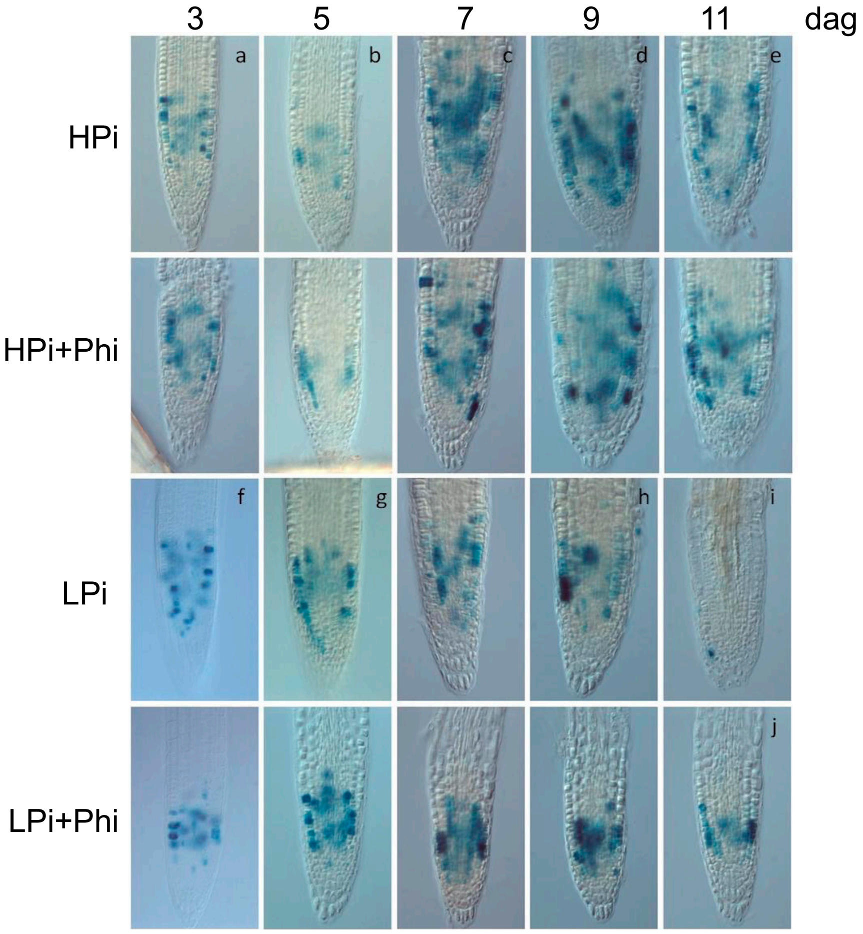

Figure S4. Effect of phosphite (Phi) on mitotic activity of the meristematic region of *Arabidopsis* CycB1;1::*uidA* seedlings. Photographs of representative meristematic regions of the primary root of *Arabidopsis* CycB1;1::*uidA* seedlings grown on agar plates under deficiency (0 M, LPi) or sufficiency (1 mM, HPi) of phosphate (Pi) supplemented with 1 mM phosphite (Phi), at different days after the germination (dag). a, b, c, d, and e, correspond to images of the meristematic zone at 3, 5,7, 9, and 11 dag under HPi; f, g, h, and i, correspond to images of the meristematic zone at 3, 5,9, and 11 dag under LPi; j corresponds to the meristematic zone at11 dag under LPi+Phi.

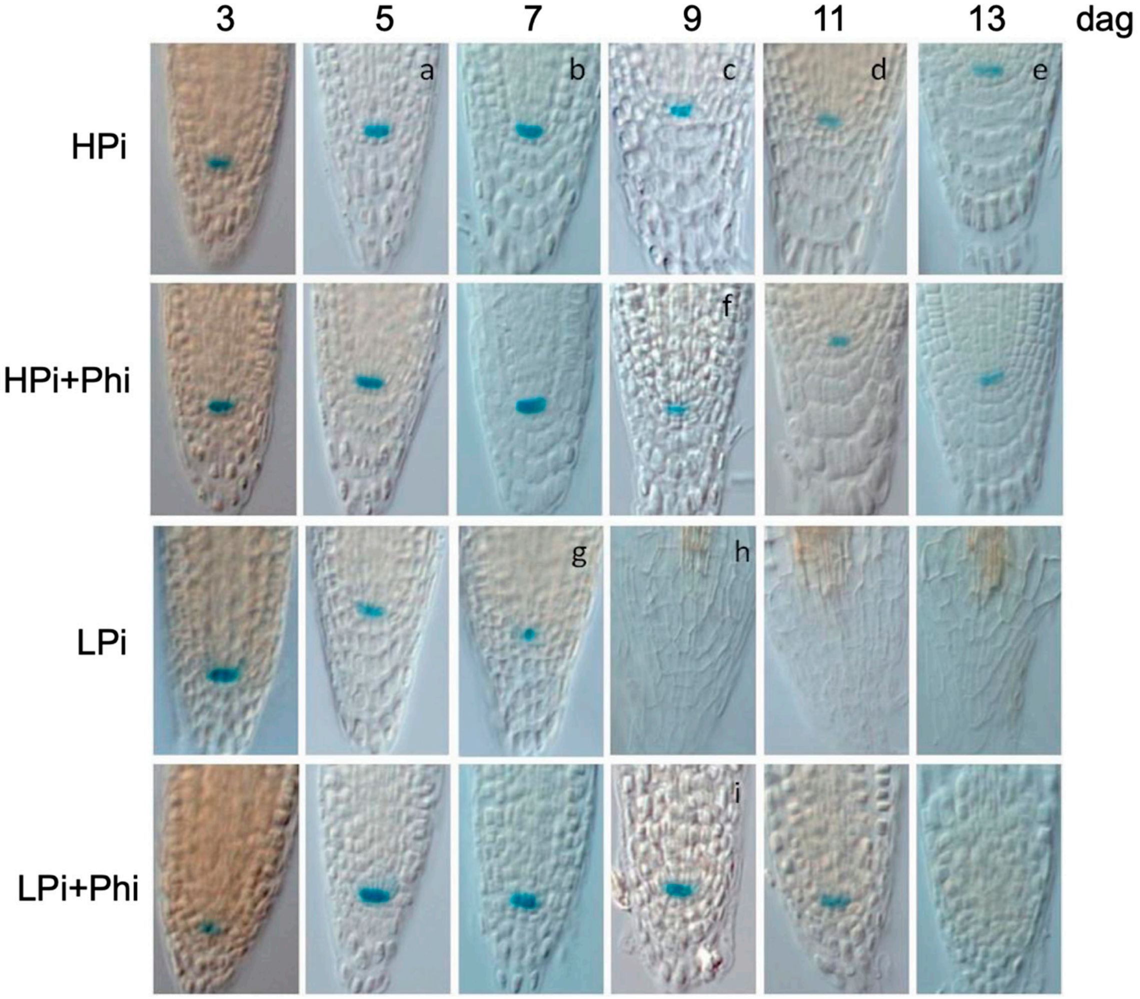

Figure S5. Effect of phosphite (Phi) on the activity of the quiescent center (QC) of *Arabidopsis* QC46::*uidA* seedlings. Photographs of representative meristematic regions of the primary root of *Arabidopsis* QC46::*uidA* seedlings grown on agar plates under deficiency (0 M, LPi) or sufficiency (1mM, HPi) of phosphate (Pi) supplemented with 1 mM phosphite (Phi), at different days after the germination (dag). The meristematic regions were photographed using the same magnification. a, b, c, d, and e, correspond to images of the meristematic zone at 3, 5,7, 9, 11, and 13 dag under HPi; f shows the meristem at 9 dag under HPi+Phi; g and h show the meristem at 7 and 9 dag under LPi; i shows the meristem at 9 dag under LPi+Phi.

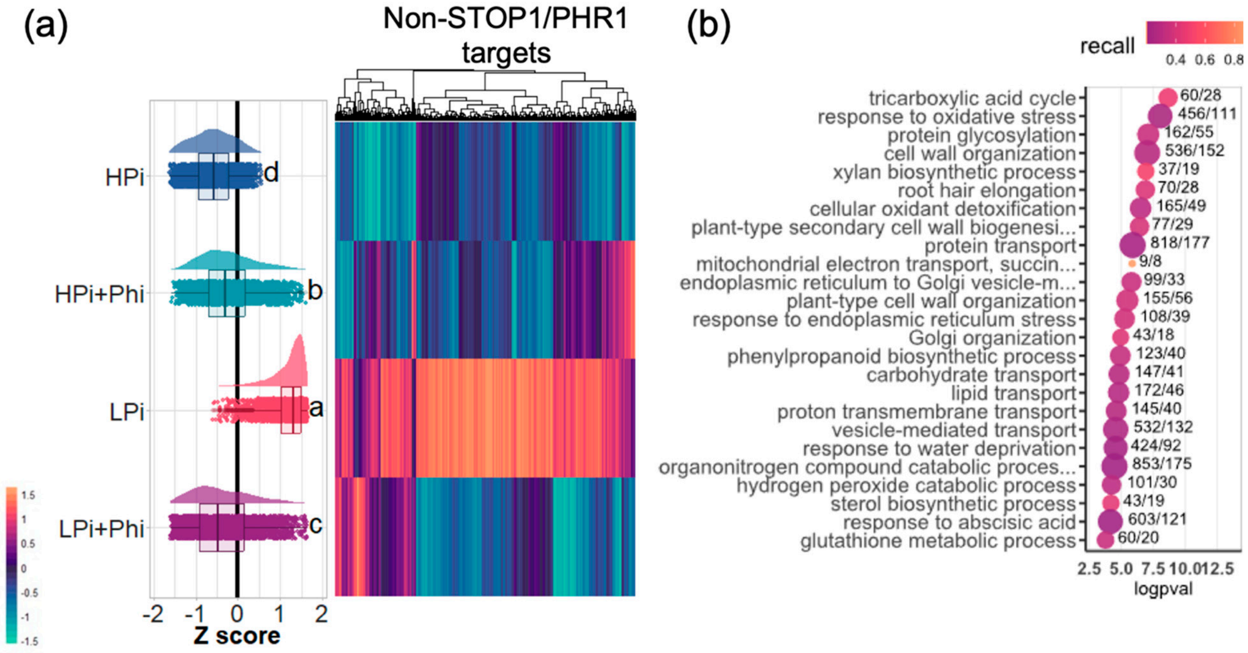

Figure S6. Effect of phosphite (Phi) on the expression pattern of non-STOP1/PHR1 target genes or genes previously described as part of the local or systemic PSRs that are upregulated in low phosphate (LPi) treatments. (a) Heatmap and rain cloud plots and (b) GO terms enrichment analysis of the non-STOP1/PHR1 target genes.

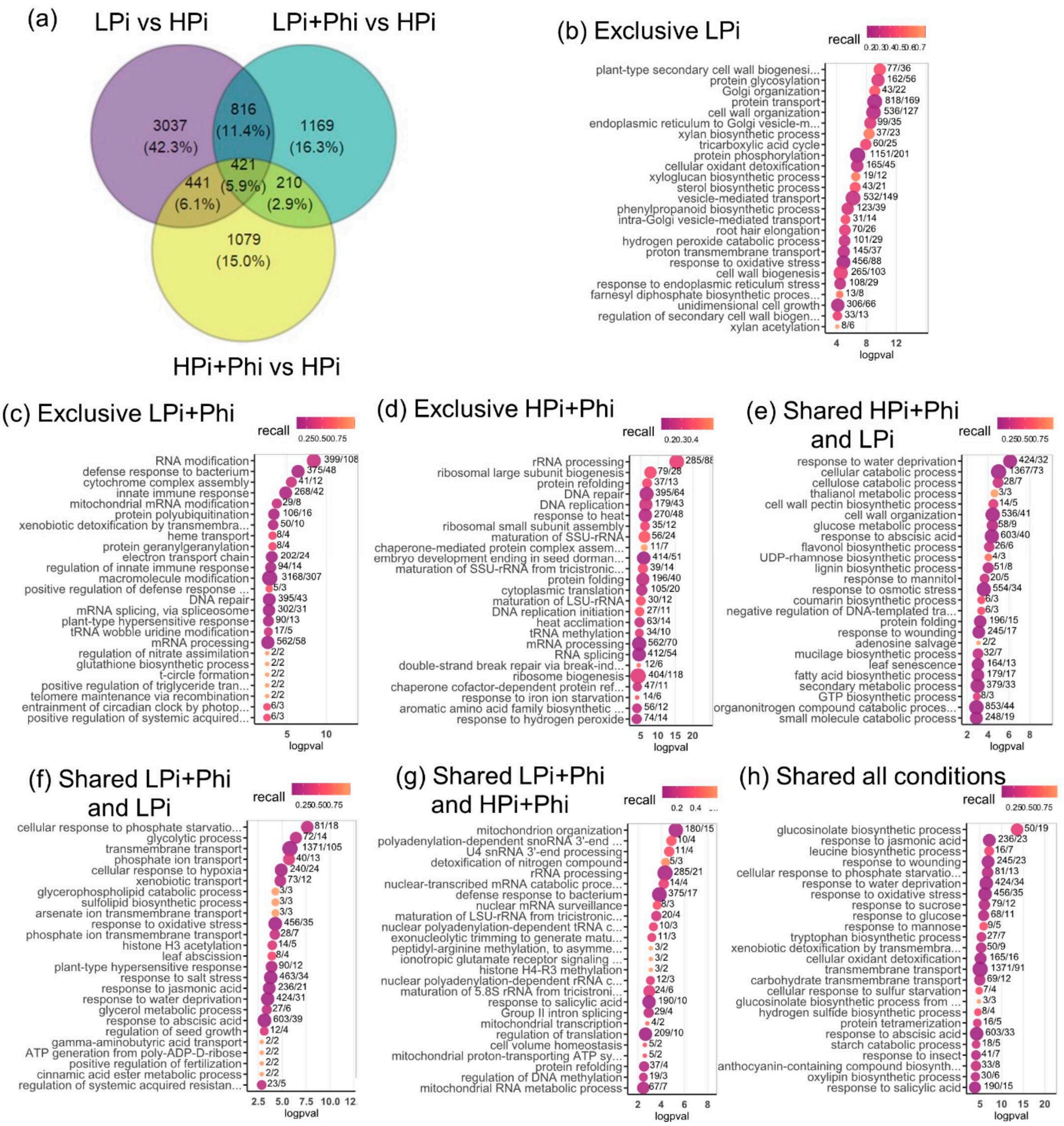

Figure S7. Effect of phosphite (Phi) on the transcriptional profile of *Arabidopsis* under the different treatments relative to the control with high phosphorus level (HPi). Each number refers to a specific set of enriched terms of the Gene Ontology. (a) Venn diagram showing the intersections of differentially expressed genes (DEGs) in all treatments. (b-g) Gene ontology (GO) terms shared and exclusive to the different treatments, and (h) GO terms shared between all conditions.
